# The role of focal adhesion anchoring domains of CAS in mechanotransduction

**DOI:** 10.1038/srep46233

**Published:** 2017-04-13

**Authors:** Jaroslav Braniš, Csilla Pataki, Marina Spörrer, Richard C. Gerum, Astrid Mainka, Vladimir Cermak, Wolfgang H. Goldmann, Ben Fabry, Jan Brabek, Daniel Rosel

**Affiliations:** 1 BIOCEV at Faculty of Science, Charles University in Prague, Vestec, Czech Republic; 2 Biophysics Group, Department of Physics, University of Erlangen-Nuremberg, Erlangen, Germany

## Abstract

CAS is a docking protein, which was shown to act as a mechanosensor in focal adhesions. The unique assembly of structural domains in CAS is important for its function as a mechanosensor. The tension within focal adhesions is transmitted to a stretchable substrate domain of CAS by focal adhesion-targeting of SH3 and CCH domain of CAS, which anchor the CAS protein in focal adhesions. Mechanistic models of the stretching biosensor propose equal roles for both anchoring domains. Using deletion mutants and domain replacements, we have analyzed the relative importance of the focal adhesion anchoring domains on CAS localization and dynamics in focal adhesions as well as on CAS-mediated mechanotransduction. We confirmed the predicted prerequisite of the focal adhesion targeting for CAS-dependent mechanosensing and unraveled the critical importance of CAS SH3 domain in mechanosensing. We further show that CAS localizes to the force transduction layer of focal adhesions and that mechanical stress stabilizes CAS in focal adhesions.

CAS (Crk associated substrate, also called p130Cas) together with EFS/SIN, HEF1/NEDD9, and CASS4 belongs to the CAS family of docking/adaptor proteins^1^ and plays a central role in the integrin-mediated regulation of cell behavior. CAS acts as a primary force sensor in focal adhesions, transducing mechanical forces into cellular response^2^. The unique assembly of structural domains of CAS is important for its function in focal adhesions and mechanosensing. The N-terminus contains of a SH3 domain that is responsible for the interaction with the poly-proline motifs including tyrosine kinases FAK, PYK2^3^,^4^, tyrosine phosphatases PTP1B, PTP-PEST^5^,^6^, and other proteins such as C3G, vinculin, or CIZ^7^,^8^,^9^,^10^. The SH3 domain was also identified as one of the two domains, which enables CAS localization in focal adhesions^11^. The ability of the CAS SH3 domain to bind ligands can be regulated by Src-mediated phosphorylation of tyrosine 12, which inhibits its binding^12^,^13^. In the central region of CAS are the substrate (SD) and serine rich (SRD) domains. The hallmark of the large central SD domain of CAS are the 15 YxxP tyrosine motifs^14^. These motifs are phosphorylated by Src family kinases during cell adhesion and increased mechanical stress^15^,^16^,^17^. SD represents the mechanosensory domain of CAS. Mechanical stretch leads to structural changes in SD, and the exposure of cryptic tyrosines are subsequently phosphorylated by Src-family kinases. Once phosphorylated, this domain serves as a docking site for SH2 domains of Crk or Nck docking proteins^14^,^18^. The C-terminus of the protein can then be divided into two parts: Src binding domain (SBD) and C-terminal CAS-family homology (CCH) domain. SBD contains binding sites for the SH2 and SH3 domain (YDYV and RPLPSPP, respectively) of Src family kinases^19^. The CCH domain is located at the C-terminus of CAS and along with the SH3 domain functions as the focal adhesion targeting domain^11^. The CCH domain shares sequential and structural characteristics with focal adhesion targeting (FAT) domains of FAK, including a four-helical bundle fold^20^.

Focal adhesions are formed at the interface of the cell surface and extracellular matrix and link the extracellular matrix to the intracellular cytoskeleton. However, focal adhesions are not passive anchors, rather they represent dynamic sites of sensing and bi-directional transmission of chemical and mechanical cues between cells and surrounding extracellular matrix. Focal adhesions are the primary sites of sensing mechanical tension in adherent cells^21^. In response to extracellular matrix rigidity or mechanical stress, they modulate the molecular composition and activity of signaling proteins^22^. The mechanical tension is sensed within the focal adhesions by primary mechanosensors, which are proteins that respond to mechanical stress by changing their conformation^2^. CAS represents one of such a primary mechanosensor in focal adhesions.

It is the unique domain structure of CAS, which allows it to act as a mechanosensor. The stretchable SD functions as a tension sensor^16^. In order to transmit tension on the SD, CAS possesses two focal adhesions-anchoring domains that are flanking the stretchable SD domain: SH3 on the N-terminus, and CCH domain on the C-terminus^11^,^16^. Mechanistic models of stretching the biosensor propose equal roles for both anchoring domains. In CAS, however, both anchoring domains differ in their structure and most probably in their binding partners. To determine the function and relative importance of the CAS anchoring domains for the mechanosensing properties of CAS, we analyzed a series of CAS constructs where the anchoring domains were either deleted or replaced with either a strong focal adhesion targeting motif of the FAT domain or in the case of the CCH domain also with a LeuZip dimerization motif.

In this work, we describe the relative importance of focal adhesion anchoring domains of CAS on CAS localization and dynamics in focal adhesions as well as CAS-mediated mechanotransduction.

## Results

### Relative importance of anchoring domains on CAS localization in focal adhesions

**Figure 1:**
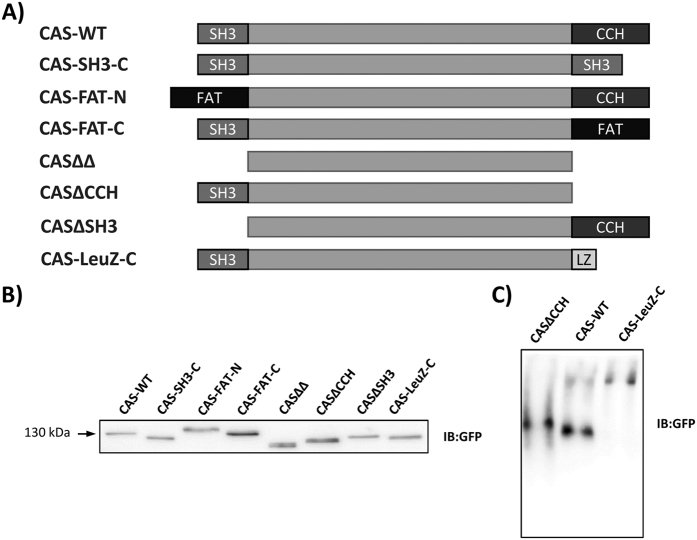
(**A**) Schematic depiction of CAS constructs. The CAS−/− cells were transfected to express GFP-fused CAS variants. (**B**) Immunoblot analysis of whole cell lysates shows expression levels of the GFP-fused CAS variants separated by SDS-PAGE and detected by anti-GFP antibody. (**C**) Immunoblot analysis of immunoprecipitated GFP-fused CAS variants separated by native PAGE and detected by an anti-GFP antibody.

To determine the relative importance of the position of the anchoring domains within the structure of CAS and their exclusivity for the mechanosensing properties of CAS, we designed a series of mutant variants of CAS and expressed them in CAS−/− MEFs (Fig. 1A,B). We prepared CAS constructs tagged with GFP on the N-terminus, where the anchoring domains were individually or completely deleted (CASΔSH3, CASΔCCH, and CASΔΔ) or individually replaced by the focal adhesion targeting domain (FAT) of FAK (CAS-FAT-N and CAS-FAT-C)^23^. Furthermore, to test the possibility that the CCH domain functions as a dimerization domain^24^, the CCH domain was replaced by a LeuZip dimerization motif of GCN4 (CAS-LeuZ-C)^25^. In this case, the mechano-reception of CAS could be achieved by tail to tail dimerization and SH3-mediated anchoring on both ends of the dimer^2^. We also prepared a CAS construct, where the CCH domain was replaced by a CAS-SH3 domain (CAS-C-SH3), a construct mimicking a shorter version of the potential CCH-mediated CAS-CAS dimer. To confirm that the introduction of LeuZip to CASΔCCH induces its dimerization, we analyzed the mobility of precipitated CAS-WT, CAS-LeuZ-C, and CASΔCCH variants (Supplement Figure S1A) using native PAGE. We found that both CAS-LeuZ-C and CASΔCCH migrate on native PAGE as single bands; the CAS-LeuZ-C showing slower mobility (Fig. 1C). CAS-WT, however, migrates as two separate bands. The predominant lower band exhibits similar mobility as the single band of CASΔCCH, whereas the less abundant higher band shows mobility similar to the single band of CAS-LeuZ-C (Fig. 1C). Similar pattern of bands can be observed on native PAGE probed with an anti-phospho-CAS antibody, suggesting that CAS phosphorylation is not responsible for the mobility shift of both CAS-LeuZ-C and of the upper band of CAS-WT (Supplement Figure S1B). These data indicate that, when introducing the LeuZip domain to CAS, it effectively induces dimerization of CAS. In addition, the data suggest that CAS, like NEDD9^24^, can form homodimers and that CCH domain is required for dimerization.

**Figure 2:**
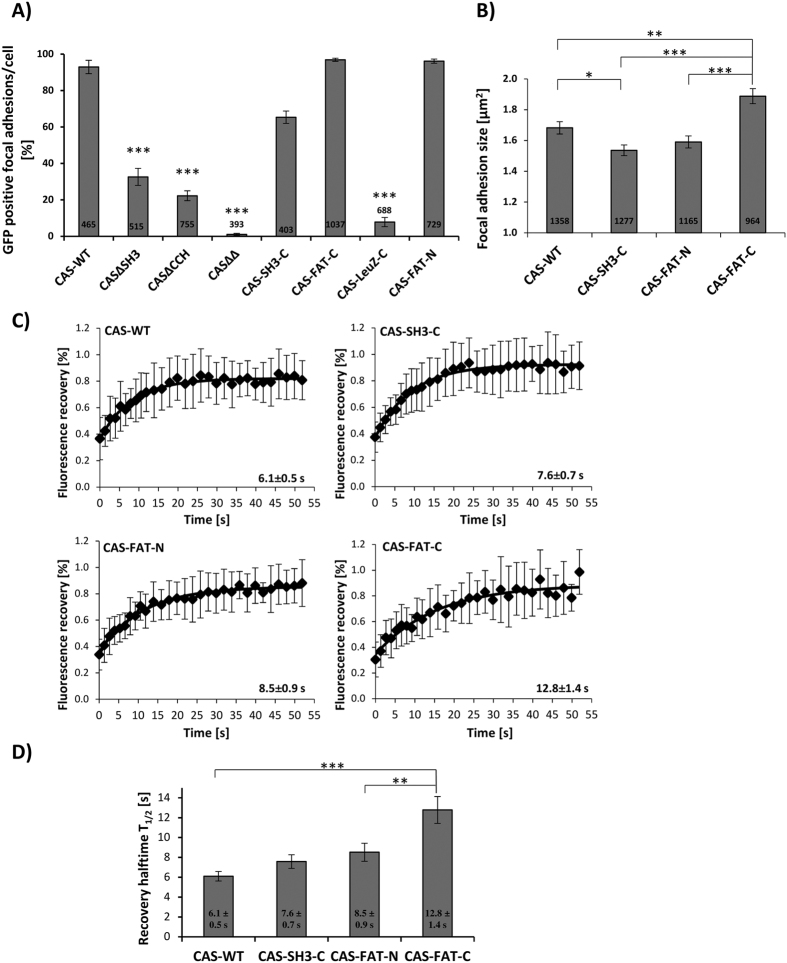
Analysis of CAS variants in focal adhesions. (**A**) The CAS−/− cells were transfected to transiently express GFP-fused CAS variants and analyzed for GFP-CAS localization in focal adhesions by confocal microscopy. The bar graph shows the percentage of CAS-positive focal adhesions. Focal adhesions were considered CAS positive if the GFP-CAS signal was at least double the signal in the cytoplasm, adjacent to the focal adhesions, indicated by paxillin staining. Asterisks indicate statistical significance compared to CAS-WT. (**B–D**) CAS−/− cells were transfected to stably express GFP-fused CAS variants and analyzed for the size of focal adhesions and GFP-CAS dynamics in focal adhesions. (**B**) The bar plot represents the focal adhesion size calculated from the paxillin signal. The numbers in the columns indicate the number of analyzed focal adhesions, error bars represent standard error of the mean (**A**,**B**). (**C**) FRAP curves of indicated CAS variants. The average recovery halftime, T_1/2_ is shown +/− standard errors. (**D**) The bar plot shows the average recovery halftime, T_1/2_ for individual CAS variants. Error bars represent standard error of the mean. Statistical significance (*p < 0.05, **p < 0.01, ***p < 0.001) was determined by one-way ANOVA followed by Tukey’s post-hoc test.

The GFP-tagged CAS variants were further analyzed for their localization in focal adhesions (Fig. 1B). The co-localization study of CAS variants with the focal adhesion marker protein paxillin confirmed that the SH3 and CCH domain are important for anchoring the molecule in focal adhesions, whilst the double mutant (CASΔSH3ΔCCH) is almost totally missing in focal adhesions (Fig. 2A). Replacement of the intrinsic CAS anchoring domains with the FAT domain completely rescues the focal adhesion targeting of these variants and confirms that the FAT domain is able to fulfil the function of these domains for focal adhesions targeting. The CAS-LeuZ-C variant localized poorly in focal adhesions, showing that forced tail to tail dimerization of CAS with the deleted CCH domain cannot rescue it for focal adhesion targeting. Interestingly, however, the replacement of the CCH domain with SH3 resulted in almost complete rescue of CAS localization in focal adhesions (Fig. 2A).

We further investigated the size of focal adhesions in cells expressing CAS variants, which show a high degree of FA localization. The expression of the CAS-FAT-C variant results in significantly larger focal adhesions compared to WT, while the cells with the CAS-C-SH3 variant have smaller focal adhesions than WT (Fig. 2B).

### The effect of different modes of CAS anchoring in focal adhesions on CAS dynamics, signaling, and migration

The differences in the level of CAS constructs localization in focal adhesions and in the effect on size of focal adhesions suggests contrasting degrees of CAS dynamics in focal adhesions. We focused on CAS variants, which are effectively localized in focal adhesions, and analyzed the effect of their expression on CAS dynamics in focal adhesions using FRAP. We found that the CAS-FAT-C variant was significantly more stable in focal adhesions then WT. The CAS-FAT-N variant also showed a trend to increased stability in focal adhesions; however, it was not statistically significant (Fig. 2C,D). These results show that replacing CAS anchoring domains with the strong focal adhesion targeting signal (FAT domain) leads to the stabilization of CAS in focal adhesions, which is more prominent when the CCH and not the SH3 domain is replaced.

**Figure 3:**
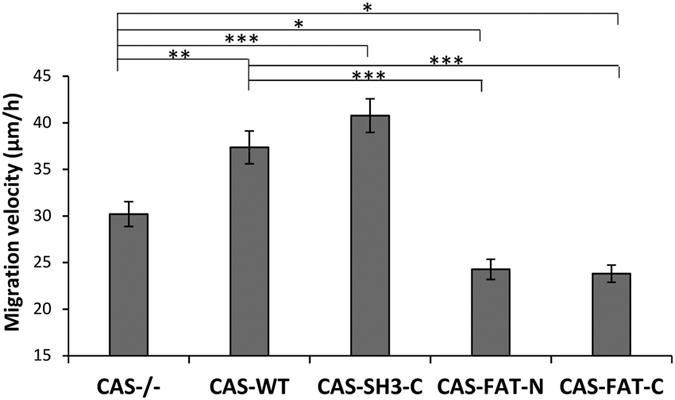
Presence of FAT domain significantly reduces migration velocity. The CAS−/− cells stably expressing GFP-fused CAS variants were analyzed for migration, using a wound healing assay. Migration was quantified by tracking nuclei and calculated as the total distance of cells migrating over 2–8 hours after wounding. Error bars represent standard error of the mean. Statistical significance (*p < 0.05, **p < 0.01, ***p < 0.001) was determined by one-way ANOVA followed by Tukey’s post-hoc test.

Focal adhesion size and dynamics are often correlated with the migratory behavior of cells. We, thus analyzed the effect of the CAS variants on migration, using a wound healing assay. A wound scratch was introduced in confluent monolayers of the cells using a pipette tip, and the mean velocity of the cells closing the wound was determined. Consistent with changes in the dynamics of CAS localization to focal adhesion, we found that expression of CAS-FAT-N and CAS-FAT-C in CAS−/− cells slows down the cell migration compared to the expression of CAS-WT or CAS-C-SH3 (Fig. 3).

**Figure 4:**
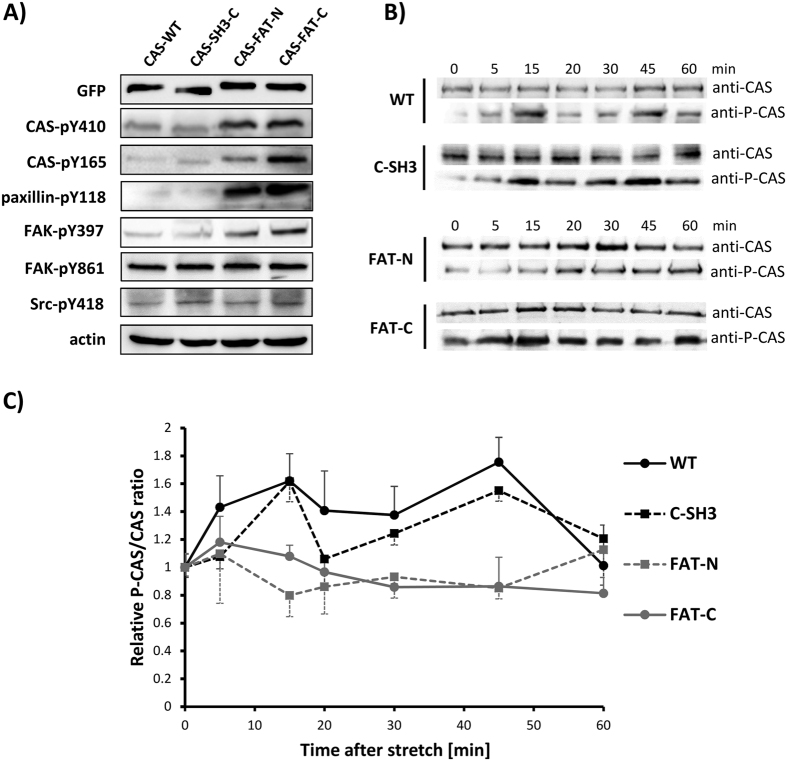
The effect of replacement of focal adhesion anchoring domains on CAS-related signaling and mechanotransduction. (**A**) The CAS−/− cells stably expressing GFP-fused CAS variants were analyzed for expression or phosphorylation of indicated proteins by immunoblotting. (**B**,**C**) The CAS−/− cells stably expressing GFP-fused CAS variants were plated on a stretchable PDMS membrane coated with fibronectin, incubated overnight and then subjected to 20% static stretch at indicated times. (**B**) Immunoblot analysis of phosphorylation dynamics of indicated CAS variants on substrate domain after 20% static stretch. Representative images are shown. (**C**) Quantification of relative phospho-CAS and CAS Western blot signals. Time points represents averages calculated from a minimum 3 independent experiments; error bars represent the standard deviation.

Our data indicate that substituting individual anchoring domains of CAS with a FAT or CCH domain has almost no effect on CAS targeting to focal adhesions. However, introducing these substitutions leads to significantly altered focal adhesion size, dynamics of CAS in focal adhesions, and also to changes in cellular migration. This suggests that CAS SD-phosphorylation-dependent signaling is significantly affected by these changes. Indeed, immunoblotting analysis revealed that in variants, where the SH3 or CCH domains are replaced by the FAT domain, the phosphorylation of CAS-SD is significantly increased compared to WT or that the SH3 domain was inserted in the C-terminus instead of CCH (CAS-C-SH3). The higher SD phosphorylation levels were also followed by higher paxillin phosphorylation (Y118) and higher FAK phosphorylation on position Y397. However, phosphorylation levels of FAK on Y861 and Src on Y418 are similar in all variants tested, indicating that the activation status of Src is comparable in all these variants (Fig. 4A).

Besides basal levels of CAS-related signaling in resting cells, we also analyzed the effect of mechanical stretch of cells on the phosphorylation of the SD domain of CAS. We subjected cells with different CAS mutant variants to a 20% static stretch and checked the dynamics of SD domain phosphorylation after the stretch. The WT and CAS-SH3-C mutant that possess two SH3 domains responded similarly to mechanical stimulation with two phosphorylation peaks at 15 and 45 minutes after stretch (Fig. 4B,C). The mutants, in which the SH3 or CCH domains were replaced by the FAT domain, showed no significant fluctuation in SD domain phosphorylation after stretch. This, however, could be due to the already high basal phosphorylation of SD domain, which could effectively mask a milder stretch-induced increase of the phosphorylation (see Fig. 4A).

### The effect of CAS anchoring domains replacement on traction forces and adhesion strength

The observation of changes in focal adhesion size, CAS dynamics in focal adhesions, and the effects on CAS-related signaling suggest that cells with different CAS variants differ in adhesion strength and traction forces. We analyzed the effect of all CAS variants on force generation using traction force microscopy^26^. Using traction force microscopy allows the determination of contractile forces and strain (deformation) energy that cells exert on their surroundings.

**Figure 5:**
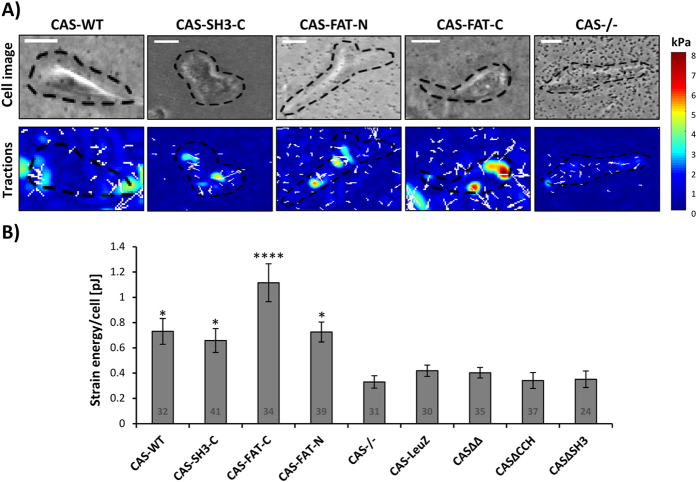
Measurements of cellular traction forces by 2D traction microscopy. The CAS−/− cells were transfected to transiently expressed GFP-fused CAS variants and analyzed using traction force microscopy. (**A**) Bright field (upper row) and traction field images (lower row) of representative CAS−/− cells, expressing indicated variants of CAS. (**B**) The bar plot shows the strain energy generated by individual cells. Numbers in columns indicate number of analyzed cells, and the error bars represent the standard error of the mean. Statistical significance comparing individual variants to CAS−/− cells was determined by one-way ANOVA followed by Tukey’s post-hoc test (*p < 0.05, ****p < 0.0001).

We observed that CAS−/− cells expressing CAS variants, which are effectively localized in focal adhesions (CAS-WT, CAS-FAT-C, CAS-FAT-N, and CAS-C-SH3), exhibit significantly higher traction forces than the parental CAS−/− cells. The highest traction forces and statistical significance over CAS−/− were exhibited by the CAS-FAT-C expressing cells (Fig. 5A). Interestingly, CAS-FAT-C cells generated significantly higher (p = 0.021) traction forces than CAS-FAT-N. This suggest that besides stabilization of CAS in focal adhesions and enhanced CAS-related signaling, the presence of the CAS-SH3 domain, missing in CAS-FAT-N variants, is necessary for effective signaling that leads to the generation of traction forces. In addition to CAS variants, which are effectively localized to focal adhesions, we also analyzed other CAS variants introduced in Fig 1. None of these were able to rescue the decreased strain energy in CAS−/− cells (Fig. 5B). This, further emphasizes the importance of CAS localization to focal adhesions in cellular force generation.

**Figure 6:**
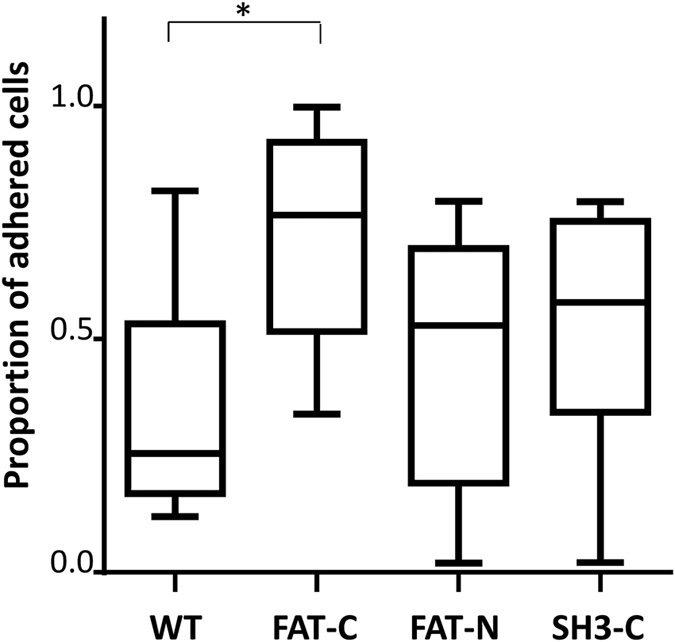
The effect of CAS variants on cellular adhesion strength. Approximately 15,000 cells of CAS−/− cells stably expressing GFP-fused CAS variants were seeded in a Petri dish. Next day, the Petri dish was placed into the spinning disk shear rheometer and the shear stress of 0.7–1.5 Pa was introduced for 5 minutes by rotation of the inserted transparent rotating plate. The number of cells before and after the shear was counted in the same area of the plate, 2–4 mm from the center of the dish. The box-and-whisker graph shows the range in ratio of number of cells in the analyzed area after and before the spin. The center line shows the median, box limits indicate the first and third quartile, whiskers extend to minimum and maximum. The data were statistically analyzed with quasi-binomial generalized linear model (*p < 0.05).

The difference in traction forces could be indicative of different adhesion strength. We, thus analyzed the cellular adhesion strength using a spinning disk shear rheometer. This device is equal to a rotational parallel plate viscometer with a few modifications and consists of a stationary and a rotating plate, which generates a shear flow and consequently a shear stress on cells bound to the stationary plate. We found that, indeed the CAS-FAT-C expressing cells (similar to strain energy, Fig. 5B), exhibit significantly higher adhesion strength when compared to other CAS variants (Fig. 6).

### Mechanical stress stabilizes CAS in focal adhesions

**Figure 7:**
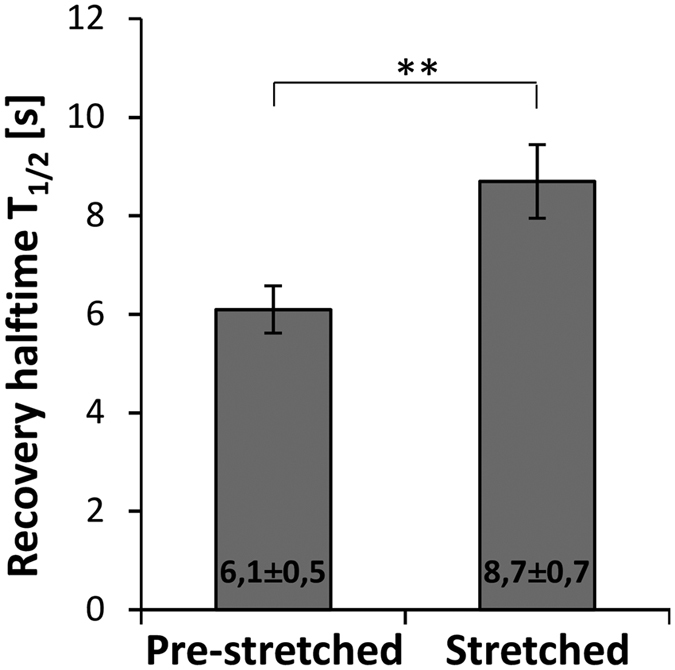
Effect of cellular stretching on CAS dynamics within focal adhesions. CAS−/− cells stably expressing GFP-fused CAS-WT were plated on a stretchable PDMS membrane coated with fibronectin, incubated overnight and then subjected to 20% static stretch during FRAP measurement. The bar graph shows the average recovery halftime, T_1/2_ for GFP-CAS-WT. The column “Pre-stretched” represents FRAP measurements in quiescent cells. The column “Stretched” represents FRAP measurement of GFP-CAS-WT in stretched cells within the period of 30 min after stretch. Error bars represent standard error of the mean. Statistical significance (**p < 0.01) was determined by one-way ANOVA.

The CAS variants, where the CCH domain was replaced by the FAT domain (CAS-FAT-C), have larger focal adhesions and exhibit slower CAS dynamics in focal adhesions as well as the highest traction forces. From these results, we hypothesized that increased cellular traction forces stabilize CAS in focal adhesions. To test that, we subjected the cells to a 20% static stretch and analyzed the CAS dynamics in focal adhesions. Indeed, the CAS dynamics decreased significantly (Fig. 7). This confirms that mechanical tension, both external and intracellular, affects CAS dynamics in focal adhesions.

### The insertion of FAT domain localizes CAS to a lower layer of focal adhesions

**Figure 8:**
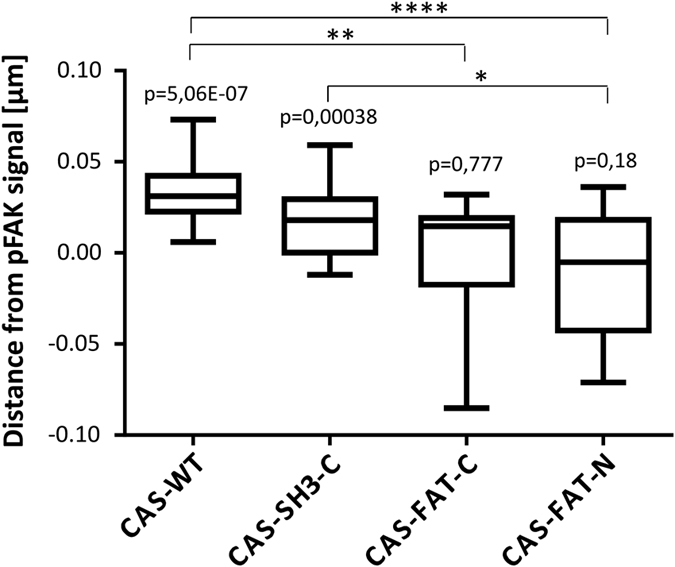
Nanoscale localization of CAS variants in focal adhesions. CAS−/− cells stably expressing GFP-fused CAS variants were analyzed for vertical distribution of fluorescence intensity signal of CAS variants and phospho-FAK (pFAK) in focal adhesions. Box-and-whisker plot shows the range in the difference between indicated CAS variants and pFAK vertical peaks (z-centers). P-values above the boxes indicate statistical significance in the localization of z-centers of pFAK and corresponding CAS signals within individual focal adhesions determined by paired T-tests. Asterisks brackets indicate significance (*p < 0.05, **p < 0.01, ****p < 0.0001) in nanoscale localization of the individual CAS variants determined by one-way ANOVA.

Within the 3D nanostructure of focal adhesions three functional and topographical layers were defined. From bottom to top, it is the plasma membrane apposed integrin signaling layer, in the middle is a force transduction layer, and on the top is an actin regulatory layer^27^. Our data show that, while the replacement of the intrinsic CAS anchoring domains with the FAT domain completely rescues the focal adhesion targeting, it also inhibits the migratory behavior of cells and CAS-mediated mechanotransduction. The dominant effect of the FAT domain for cell migration and CAS-mediated mechanotransduction could be indicative of the mislocalization of these variants to a non-physiological layer of focal adhesions. We, thus analyzed the localization of the CAS variants within the nanoarchitecture of focal adhesions using a 3D STED super-resolution microscope. To compare between the CAS variants, the phospho-FAK (P-Y397) signal was used as a marker for the integrin signaling layer and as reference control. The x/z intensity profiles of GFP-CAS variants and phospho-FAK signal in individual focal adhesions were acquired using the 3D STED in fixed cells, fitted by a Gaussian fit, and the center of vertical positions (z-center) were determined from the Gaussian fit to the molecule peak. We found that the peak of vertical distribution of CAS-WT and CAS-C-SH3 variants is significantly different from phospho-FAK. On average, CAS-WT is approximately 30 nm higher than phospho-FAK, suggesting that CAS localizes in the force transduction layer. In contrast, the peak of the vertical distribution of both CAS variants with inserted a FAT domain is similar (not significantly different) to that of phospho-FAK, suggesting mislocalization of these variants to the integrin signaling layer (Fig. 8, Supplement Figure S2).

## Discussion

Using deletion mutants and domain replacements, we have analyzed the relative importance of focal adhesion anchoring domains of CAS on CAS localization and dynamics in focal adhesions as well as CAS-mediated mechanotransduction. We have confirmed observations by others that SH3 and CCH domains are the main focal adhesion targeting domains^11^. Both intrinsic anchoring domains of CAS can also be effectively replaced by the FAT domain for targeting of CAS to focal adhesions and for traction force generation. However, for dynamics of CAS phosphorylation after mechanical stretch and cell migration, the replacement of the intrinsic anchoring domain of CAS with FAT cannot rescue the CAS−/− phenotype. This suggests that in mechanotransduction neither the SH3 nor CCH domain function as a simple focal adhesion targeting sequence.

Replacement of either anchoring domains of CAS by FAT greatly increases the tyrosine phosphorylation status of CAS, paxillin on Y118, and FAK on Y397. The position Y397 represents an autophosphorylation site of FAK and its phosphorylation exposes a SH2 domain-binding site for Src^28^. Phosphorylation of Y397 is not, however, followed by an increase in phosphorylation of Y861, a major Src phosphorylation site of FAK^29^. Consistently with low phosphorylation of FAK on Y861, the auto-phosphorylation in the activation loop of Src (Y418) is also not enhanced in CAS variants with the FAT domain, suggesting that Src activation status is similarly affected by all CAS variants tested. This observation is, however, in contrast with increased CAS and paxillin phosphorylation. These data suggest that FAK could directly phosphorylate these two CAS variants. Interestingly, we also found that CAS variants with the FAT domain, unlike CAS-WT and CAS-C-SH3, localize to the same layer of stratified structure of focal adhesions as FAK. Potentially, this could greatly enhance the ability of FAK to directly phosphorylate CAS variants with the FAT domain.

The replacement of the FA anchoring domains of CAS by FAT enhances the FAK-mediated signaling; however, the outcome of such increased signaling greatly differs between the FAT-N and FAT-C variants. FAT-C expressing cells exhibited increased adhesion strength and generated higher traction forces, suggesting that the presence of the SH3 domain in FAT-C variant is important for transmitting the increased activation status of FAK and higher CAS phosphorylation. The critical importance of SH3 domain in CAS-dependent processes is further supported by the behavior of the CAS-SH3-C mutant, which despite the suboptimal localization in focal adhesions, effectively restores the ability of cells to respond to the mechanical stretch and generate traction forces.

The critical importance of CAS-SH3 domain in processes dependent on anchoring of CAS in focal adhesions could be related to an action of one or many of its diverse interacting partners^2^. Alternatively, it was shown that the interaction of the CAS-SH3 domain with its substrates can be regulated by phosphorylation on Tyr12^9^,^12^,^13^. This enables dynamically regulated changes in CAS targeting to focal adhesions, which might be critical for proper subsequent signaling.

With the help of the CAS variant, where the CCH domain was replaced with LeuZip dimerization motif (CAS-LeuZ-C), we have shown that CAS can dimerize and that this dimerization is CCH domain-dependent. However, this forced homo-dimerization of CAS does not lead to effective localization of this variant to focal adhesions. In fact, its ability to localize to focal adhesions is even lower than that of its parental CASΔCCH variant (p = 0.024). In addition, the CAS-LeuZ-C cannot rescue the CAS−/− phenotype with traction forces. Together, these data indicate that though dimerization might play an important role in CAS physiology, it is probably inhibitory for CAS localization to focal adhesions and may not play a direct role in CAS-mediated induction of traction forces. Thus, CCH-dependent targeting of CAS to focal adhesions is not due to dimerization, but rather to direct interaction of some integral protein(s) of focal adhesions.

The use of the chimeric variants of CAS proved to be a valuable approach for in-depth analyses in CAS protein biology. We have unraveled the critical importance of the SH3 domain in CAS-dependent mechanosensing and mechanotransduction. For the first time, we have shown that CAS like HEF1 can dimerize. We have also analyzed the localization of the CAS variants within the nano-architecture of focal adhesions and demonstrated that CAS localizes significantly higher in focal adhesions than phospho-FAK, which is consistent with CAS localization to the force transduction layer^27^. Finally, we have shown that mechanical stress stabilizes CAS in focal adhesions.

## Materials and Methods

### Cell culture and transfection

CAS−/− MEFs were obtained from Steven Hanks (Vanderbilt University, Nashville). CAS−/− MEFs stably re-expressing CAS variants (WT, SH3-C, FAT-N and FAT-C) were prepared by using pMSCV-puro-CAS vector variants conjugated with GFP. The resulting vectors, after sequence verification, were transfected into Phoenix E packaging cells, viral supernatant were prepared and used to infect CAS−/− MEFs. MEFs stably expressing the GFP-CAS variants were then selected by FACS sorting for GFP. For transient transfections of CAS−/−, MEFs were transfected with CAS variants in pEGFP-C1 vectors. Transfections were carried out according to the manufacturer’s protocol, using JetPrime (Polyplus Transfection) or Lipofectamine 2000 reagent (Invitrogen). Cells were cultured in full DMEM (Sigma) with 4.5 g/l L-glucose, L-glutamine and pyruvate, supplemented with 10% fetal bovine serum (Sigma) and 2% antibiotics/antimycotics (Life Technologies) at 37 °C and 5% CO_2_.

### Wound healing assay

Cells were grown to confluence in 6-well plates coated with 5 μg/ml fibronectin (Invitrogen). A yellow pipette tip was used for scratch wound assays; the cells were then washed with 1 × PBS and full DMEM (Sigma). Cells were cultured at 37 °C with 5% CO_2_. Cell migration was visualized on a JuLI FL live cell imager. Time-lapsed images of cells were captured every 2 min for 8 hours. To determine total migration distance for each cell, nuclei of 45 cells (15 each from three independent experiments) migrating into the scratch wound were tracked, using ImageJ (National Institutes of Health, Bethesda, MD) MTrackJ plugin. The mean velocity was calculated for each cell population over 6 h (two to eight hours post wounding). Only non-dividing cells were analyzed.

### Cell stretching

Cell stretching experiments were carried out on 10 cm^2^ stretchable PDMS chambers (STREX Inc.). The substrates of chambers were coated with 5 μg/ml fibronectin (Invitrogen). Cells were seeded on fibronectin-coated substrates and cultured overnight at 37 °C with 5% CO_2_. Uniaxial static stretching was performed in the incubator under regular cell culture conditions, using a manual stretcher (STREX Inc.). Cells were stretched for various time intervals (0 min, 5 min, 15 min, 20 min, 30 min, 45 min and 60 min) at an amplitude of 20% and then immediately lysed and used for immunoblotting.

### DNA constructs

All the CAS variants were constructed based on pEGFP-C1-CAS^12^. The fragment CASΔSH3 corresponding to the region 196–2622 of mouse CAS-cDNA was PCR- amplified, using the forward primer *AGATCTATGTATGATAAGAAGCCAGTAGGAC* and the reverse primer *GAATTCTCAGGCAGCAGCTAGCTGGC* and ligated BglII/EcoRI to pEGFP-C1. The CASΔCCH, corresponding to region 1–2145 of mouse CAS-cDNA was PCR amplified, using the forward primer *GGATCCATGAAGTACCTGAACGTGC* and the reverse primer *GAATTCTCAACTAGTCACCTCCTGCTCCAGTC* and ligated BamHI/EcoRI to BglII/EcoRI of pEGFP-C1. The CASΔΔ corresponding to region 196–2145 of mouse CAS-cDNA was PCR amplified, using the forward primer *AGATCTATGTATGATAAGAAGCCAGTAGGAC* and the reverse primer *GAATTCTCAACTAGTCACCTCCTGCTCCAGTC* and ligated BglII/EcoRI of pEGFP-C1. The CAS-FAT-N was constructed based on CASΔSH3 variant with FAT domain inserted to BglII site. The FAT domain of mouse FAK corresponding to region 2071–3156 was PCR amplified, using the forward primer *GGATCCATGGGTGTCAAGCTTCAGCC* and the reverse primer *GGATCCGTGTGGCCGTGTCTGC*. The CAS-FAT-C was constructed based on CASΔCCH variant with the FAT domain inserted into SpeI/EcoRI sites. The FAT domain of mouse FAK, corresponding to region 2071–3156 was PCR amplified, using the forward primer *ACTAGTGGTGTCAAGCTTCAGCC* and the reverse primer *GAATTCTCAGTGTGGCCGTGTC*. The CAS-LeuZ-C was constructed based on CASΔCCH variant with the LeuZip motif inserted to SpeI/EcoRI sites. The LeuZip motif of S. cerevisiae GCN4 corresponding to region 748–846 was PCR amplified, using the forward primer *ACTAGTATGAAACAACTTGAAGACAAGG* and the reverse primer *GAATTCTCAGCGTTCGCCAAC*. The CAS-C-SH3 was constructed based on CASΔCCH variant with SH3 domain inserted to SpeI/EcoRI sites. The SH3 domain of mouse CAS, corresponding to region 1–200, was PCR amplified using the forward primer *ACTAGTATGAAGTACCTGAACGTGC* and the reverse primer *GAATTCTACATGCCAACCAGAATC*.

### Fluorescence recovery after photobleaching (FRAP)

FRAP experiments were performed on a confocal microscope (Leica) with a 20x dip-in objective inside the heated chamber at 5% CO_2_. Transfected cells were cultured in 35 mm dishes overnight and then used for FRAP analysis. A 488 nm argon laser was used for GFP excitation and bleaching 60–70% of the fluorescence intensity in the spot. The image acquisition started 12 s before bleaching and continued for approximately 55 s (1 frame every 1.3 s). The recovery curves of the bleached regions were calculated from extracted image series, and the recovery halftime values were calculated from the FRAP curves as described by ref. 30.

### Fluorescence microscopy, focal adhesion size analysis, and 3D STED super-resolution microscopy

Transfected cells were seeded on the coverslips coated with 5 μg/ml fibronectin (Invitrogen). The day after, cells were fixed in 4% paraformaldehyde and permeabilized, using 0.3% Triton X-100 washed thoroughly with 1 × PBS and blocked in 3% bovine serum albumin in 1 × PBS. Subsequently, cells were sequentially incubated with primary antibodies for 2 h, with secondary antibodies for 1 h and between each step washed extensively with 1 × PBS.

Cells for analysis of focal adhesion size were captured by a fluorescence microscope Nikon (40x oil-immersion objective). Focal adhesion size was calculated by using ImageJ (National Institutes of Health, Bethesda, MD) as described by ref. 31. For 3D STED super-resolution microscopy, the fixed samples were prepared similar to fluorescence microscopy and mounted in Abberior Mount Liquid Antifade mounting media. The vertical localizations of phospho-FAK (P-Y397 antibody) and GFP fused CAS variants (anti-GFP antibody) in focal adhesions were analyzed, using Abberior STED 775 QUAD Scanning microscope (Abberior Instruments GmbH, Göttingen, Germany) equipped with Nikon Ti-E body, QUAD beam scanner, easy3D STED Optics Module (based on spatial light modulator, for phase pattern formation) and Nikon CFI Plan Apo Lambda objective (60x Oil, NA 1.40). Sample was illuminated by pulsed 561 nm and 640 nm lasers (40 MHz, intensities at sample position: ~20 μW) and depleted by pulsed 775 nm STED laser of 3D donut shape (40 MHz, pulse width ~800 ps, intensity at sample position: 45 mW, 100% 3D). Fluorescence signal was filtered (emission bandpass filters: 605–625 nm and 650–720 nm; pinhole 1 AU) and detected on single photon counting modules (Excelitas Technologies). To obtain the image of focal adhesions displayed parallel to the image x-axis, rotated x/z-scans were acquired (rotation <20 deg). The size of the image was dependent on the size of the structure of interest; the pixel size was fixed (20 × 50 nm). Two-color acquisition was based on line-interleaved mode with gated detection. The fluorescence intensity profiles in z-axis were extracted from microscopy data using ImageJ (National Institutes of Health, Bethesda, MD). To quantify the localizations in the focal adhesion area, the z-axis fluorescence intensity profiles were fitted by a Gaussian fit, and the center of vertical positions (z-center) was determined from a Gaussian fit to the molecule peak.

### Immunoblotting

Subconfluent cells were washed on ice with 1 × PBS and lysed, using RIPA buffer (0.15 M NaCl; 50 mM Tris-HCl, pH 7.4; 1% Nonidet P-40; 0.1% SDS; 1% sodium deoxycholate; 5 mM EDTA; 50 mM NaF). Protein concentration of lysates were determined by using the DC Protein Assay (Bio-Rad). Protein lysates were diluted in Laemmli sample buffer (0.35 M Tris-HCl, pH 6.8; 10% SDS, 40% glycerol; 0.012% bromophenol blue) with 1 mM DTT. Protein samples were separated on 10% SDS-polyacrylamide gels and transferred onto nitrocellulose membranes. The membranes were usually cut after the transfer to enable probing for up to 3 proteins of different molecular mass (e.g. 1–2 proteins of interest and a loading control). Membranes were then blocked in Tris-buffered saline (PBS), containing 5% non-fat dry milk (or 4% bovine serum albumin depending on the antibody) for 1 h at room temperature to prevent non-specific activity. After blocking, membranes were incubated overnight at 4 °C with a primary antibody, washed properly with PBS and Tween-20 (TTBS), incubated for 1 h with a secondary antibody conjugated with horseradish peroxidase. After washing with TTBS, membranes were developed by using the LAS-1000 Single System (Fujifilm).

### Immunoprecipitation and Native PAGE

Subconfluent cells were washed on ice with 1 × PBS and lysed, using Nonidet buffer (20 mM Hepes, 100 mM KCl, 0,5% Nonidet P-40, 1 mM DTT). Protein concentrations in lysates were determined using the DC Protein Assay (Bio-Rad). Lysates containing 500 mg of proteins were incubated for 2 h with 3 μg of anti-GFP antibody (mouse monoclonal antibody; Invitrogen). To prevent contamination of immuno-precipitated CAS variants with an anti-GFP antibody, the antibody was first cross-linked to ProteinA Sepharose (GE Healthcare), using Dimethyl pimelimidate. The beads were then washed with 0.2 M triethanolamine in PBS, quenched with 50 mM ethanolamine in PBS, and the unlinked antibody was removed with 1 M glycine, pH = 3. The immunoprecipitates were washed five times with ice-cold Nonidet buffer, eluted for 10 min using 0.1 M glycine (pH = 3.5) and neutralized by using 1 M Tris (pH = 9.2). Samples were diluted in 2x sample buffer (0.5 M Tris, 60% glycerol, 1% bromophenol blue) and processed for native PAGE or control SDS-PAGE.

The stacking gels for native PAGE gels contained 4% acrylamide/bis-acrylamide solution (37.5:1) in 130 mM Tris (pH = 6.8). The gradient resolving gels for native PAGE gels contained 4–15% acrylamide/bis-acrylamide (37.5:1) prepared in 322 mM Tris (pH = 8.8). The native PAGE gel was run in a running buffer (0,192 M glycine, 25 mM Tris, pH ≈ 8.3) overnight at 4 °C and 1 mA at limit. Proteins were transferred onto nitrocellulose membrane and CAS-GFP variants were detected as described above.

### 2D traction microscopy

The measurements of cellular traction forces were performed on 7% acrylamide/bisacrylamide (ratio 29:1; Sigma) gel with 18 kPa Young’s modulus with red fluorescent beads embedded at the top of the surface. The surface of the gel was activated, using Sulfo-SANPAH (Thermo Fisher Scientific) under UV light for 5 min and coated with 5 μg/ml fibronectin (Invitrogen). Transfected cells were seeded on the fibronectin-coated acrylamide/bisacrylamide gel 24 h before taking measurements and cultured at 37 °C with 5% CO_2_. Measurements were taken with an inverted microscope (DMI, Leica). Using 80 μM cytochalasin D and 0.25% Trypsin (Sigma), cellular traction forces were relaxed and embedded fluorescent beads were displaced. The bead displacements due to cell tractions are estimated with an unconstrained deconvolution algorithm, and cell tractions are computed using the Fourier transform traction cytometry method described by ref. 32. From the displacement field and the traction force, the strain energy is calculated.

### Spinning disk cellular adhesion assay

The strength of cellular adhesion was analyzed, using a home-built spinning disk shear rheometer. In brief, the shear rheometer consists of a stationary (petri dish) and a rotating transparent plate driven by air pressure generating the shear stress. The applied shear stress depends on the rotation frequency, the distance between rotational, and the stationary plate (adjustable on a micrometer scale), as well as on the amount of medium in the petri dish. In the stationary Petri dish approximately 15,000 cells were seeded in 1.5 ml medium for 24 hours, and the shear stress was applied by rotation of the inserted transparent rotating plate. The rotation frequency of the rotating plate was 1,500 rpm, the spinning time was 5 minutes, and the plate distance from the stationary petri dish holding the cells was approximately 360 μm. Photographs were taken from the middle of the dish outwards towards the rim in x-direction (seven field of views) and in y-direction (three fields of views). For the evaluation purposes, we used the program “clickpoints”^33^ to count the cells. We only included cells, which were 2–4 mm away from the center of the dish because of the highest shear. The shear stress in this area is between 0.7–1.5 Pa.

### Antibodies

Primary antibodies: pY410CAS (rabbit; Cell Signaling), pY165CAS (rabbit; Cell Signaling), pY118paxillin (rabbit; Sigma), paxillin (mouse, BD Transduction Laboratories), pY397FAK (rabbit; Invitrogen), pY861FAK (rabbit; Abgent), pY529Src (rabbit; Sigma), GFP (mouse; Invitrogen), actin (goat; Santa Cruz Biotechnology). Secondary antibodies (conjugated with horseradish peroxidase): goat anti-rabbit (Thermo Scientific), donkey anti-goat (Santa Cruz Biotechnology), rabbit anti-mouse (Abcam). AlexaFluor goat anti-mouse 594 (Molecular Probes) as a secondary antibody for immunofluorescence. For 3D STED imaging, Abberior STAR 635 (goat anti-rabbit) and Abberior STAR 580 (goat anti-mouse) were used as secondary antibodies.

### Statistical analysis

Paired T-test, one-way ANOVA with Tukey’s post-hoc test and Gaussian fit were performed, using Prism 6 software (GraphPad Software, Inc.). The spinning disk shear rheometer adherence data were analyzed according to ref. 33.

## Additional Information

**How to cite this article:** Braniš, J. *et al*. The role of focal adhesion anchoring domains of CAS in mechanotransduction. *Sci. Rep.* **7**, 46233; doi: 10.1038/srep46233 (2017).

**Publisher's note:** Springer Nature remains neutral with regard to jurisdictional claims in published maps and institutional affiliations.

## Supplementary Material

Supplementary Information

## References

[b1] Tikhmyanova N., Little J. L. & Golemis E. A. CAS proteins in normal and pathological cell growth control. Cell Mol. Life Sci. 67, 1025–1048 (2010). 19937461 10.1007/s00018-009-0213-1 PMC2836406

[b2] Janostiak R., Pataki A. C., Brabek J. & Rosel D. Mechanosensors in integrin signaling: the emerging role of p130Cas. Eur. J. Cell Biol. 93, 445–454 (2014). 25062607 10.1016/j.ejcb.2014.07.002

[b3] Li X. & Earp H. S. Paxillin is tyrosine-phosphorylated by and preferentially associates with the calcium-dependent tyrosine kinase in rat liver epithelial cells. J. Biol. Chem. 272, 14341–14348 (1997). 9162070 10.1074/jbc.272.22.14341

[b4] Polte T. R. & Hanks S. K. Interaction between focal adhesion kinase and Crk-associated tyrosine kinase substrate p130Cas. Proc. Natl. Acad. Sci. USA 92, 10678–10682 (1995). 7479864 10.1073/pnas.92.23.10678 PMC40675

[b5] Garton A. J., Burnham M. R., Bouton A. H. & Tonks N. K. Association of PTP-PEST with the SH3 domain of p130cas; a novel mechanism of protein tyrosine phosphatase substrate recognition. Oncogene 15, 877–885 (1997). 9285683 10.1038/sj.onc.1201279

[b6] Liu F., Hill D. E. & Chernoff J. Direct binding of the proline-rich region of protein tyrosine phosphatase 1B to the Src homology 3 domain of p130(Cas). J. Biol. Chem. 271, 31290–31295 (1996). 8940134 10.1074/jbc.271.49.31290

[b7] Kirsch K. H., Georgescu M. M. & Hanafusa H. Direct binding of p130(Cas) to the guanine nucleotide exchange factor C3G. J. Biol. Chem. 273, 25673–25679 (1998). 9748234 10.1074/jbc.273.40.25673

[b8] Nakamoto T. . CIZ, a zinc finger protein that interacts with p130(cas) and activates the expression of matrix metalloproteinases. Mol. Cell Biol. 20, 1649–1658 (2000). 10669742 10.1128/mcb.20.5.1649-1658.2000 PMC85348

[b9] Janostiak R. . CAS directly interacts with vinculin to control mechanosensing and focal adhesion dynamics. Cell Mol. Life Sci. 71, 727–744 (2014). 23974298 10.1007/s00018-013-1450-x PMC3901934

[b10] Goldmann W. H. Vinculin-p130Cas interaction is critical for focal adhesion dynamics and mechano-transduction. Cell Biol. Int. 38, 283–286 (2014). 24497348 10.1002/cbin.10204

[b11] Donato D. M., Ryzhova L. M., Meenderink L. M., Kaverina I. & Hanks S. K. Dynamics and mechanism of p130Cas localization to focal adhesions. J. Biol. Chem. 285, 20769–20779 (2010). 20430882 10.1074/jbc.M109.091207 PMC2898362

[b12] Janostiak R. . Tyrosine phosphorylation within the SH3 domain regulates CAS subcellular localization, cell migration, and invasiveness. Mol. Biol. Cell 22, 4256–4267 (2011). 21937722 10.1091/mbc.E11-03-0207 PMC3216652

[b13] Tatarova Z., Brabek J., Rosel D. & Novotny M. SH3 domain tyrosine phosphorylation–sites, role and evolution. PLoS. One. 7, e36310 (2012). 22615764 10.1371/journal.pone.0036310 PMC3352900

[b14] Sakai R. . A novel signaling molecule, p130, forms stable complexes *in vivo* with v-Crk and v-Src in a tyrosine phosphorylation-dependent manner. EMBO J. 13, 3748–3756 (1994). 8070403 10.1002/j.1460-2075.1994.tb06684.x PMC395286

[b15] Nojima Y. . Integrin-mediated cell adhesion promotes tyrosine phosphorylation of p130Cas, a Src homology 3-containing molecule having multiple Src homology 2-binding motifs. J. Biol. Chem. 270, 15398–15402 (1995). 7541040 10.1074/jbc.270.25.15398

[b16] Sawada Y. . Force sensing by mechanical extension of the Src family kinase substrate p130Cas. Cell 127, 1015–1026 (2006). 17129785 10.1016/j.cell.2006.09.044 PMC2746973

[b17] Shin N. Y. . Subsets of the major tyrosine phosphorylation sites in Crk-associated substrate (CAS) are sufficient to promote cell migration. J. Biol. Chem. 279, 38331–38337 (2004). 15247284 10.1074/jbc.M404675200

[b18] Schlaepfer D. D. & Hunter T. Focal adhesion kinase overexpression enhances ras-dependent integrin signaling to ERK2/mitogen-activated protein kinase through interactions with and activation of c-Src. J. Biol. Chem. 272, 13189–13195 (1997). 9148935 10.1074/jbc.272.20.13189

[b19] Nakamoto T., Sakai R., Ozawa K., Yazaki Y. & Hirai H. Direct binding of C-terminal region of p130Cas to SH2 and SH3 domains of Src kinase. J. Biol. Chem. 271, 8959–8965 (1996). 8621540 10.1074/jbc.271.15.8959

[b20] Mace P. D. . NSP-Cas protein structures reveal a promiscuous interaction module in cell signaling. Nat. Struct. Mol. Biol. 18, 1381–1387 (2011). 22081014 10.1038/nsmb.2152 PMC3230775

[b21] Chen C. S., Alonso J. L., Ostuni E., Whitesides G. M. & Ingber D. E. Cell shape provides global control of focal adhesion assembly. Biochem. Biophys. Res. Commun. 307, 355–361 (2003). 12859964 10.1016/s0006-291x(03)01165-3

[b22] Riveline D. . Focal contacts as mechanosensors: externally applied local mechanical force induces growth of focal contacts by an mDia1-dependent and ROCK-independent mechanism. J. Cell Biol. 153, 1175–1186 (2001). 11402062 10.1083/jcb.153.6.1175 PMC2192034

[b23] Hildebrand J. D., Schaller M. D. & Parsons J. T. Identification of sequences required for the efficient localization of the focal adhesion kinase, pp125FAK, to cellular focal adhesions. J. Cell Biol. 123, 993–1005 (1993). 8227154 10.1083/jcb.123.4.993 PMC2200138

[b24] Law S. F. . Dimerization of the docking/adaptor protein HEF1 via a carboxy-terminal helix-loop-helix domain. Exp. Cell Res. 252, 224–235 (1999). 10502414 10.1006/excr.1999.4609

[b25] Zeng X., Zhu H., Lashuel H. A. & Hu J. C. Oligomerization properties of GCN4 leucine zipper e and g position mutants. Protein Sci. 6, 2218–2226 (1997). 9336844 10.1002/pro.5560061016 PMC2143569

[b26] Rosel D. . Up-regulation of Rho/ROCK signaling in sarcoma cells drives invasion and increased generation of protrusive forces. Mol. Cancer Res. 6, 1410–1420 (2008). 18819929 10.1158/1541-7786.MCR-07-2174

[b27] Kanchanawong P. . Nanoscale architecture of integrin-based cell adhesions. Nature 468, 580–584 (2010). 21107430 10.1038/nature09621 PMC3046339

[b28] Schaller M. D. . Autophosphorylation of the focal adhesion kinase, pp125FAK, directs SH2-dependent binding of pp60src. Mol. Cell Biol. 14, 1680–1688 (1994). 7509446 10.1128/mcb.14.3.1680-1688.1994 PMC358526

[b29] Calalb M. B., Zhang X., Polte T. R. & Hanks S. K. Focal adhesion kinase tyrosine-861 is a major site of phosphorylation by Src. Biochem. Biophys. Res. Commun. 228, 662–668 (1996). 8941336 10.1006/bbrc.1996.1714

[b30] Tolde O., Rosel D., Janostiak R., Vesely P. & Brabek J. Dynamics and morphology of focal adhesions in complex 3D environment. Folia Biol. (Praha) 58, 177–184 (2012). 23249636 10.14712/fb2012058050177

[b31] Horzum U., Ozdil B. & Pesen-Okvur D. Step-by-step quantitative analysis of focal adhesions. MethodsX. 1, 56–59 (2014). 26150935 10.1016/j.mex.2014.06.004 PMC4472847

[b32] Butler J. P., Tolic-Norrelykke I. M., Fabry B. & Fredberg J. J. Traction fields, moments, and strain energy that cells exert on their surroundings. Am. J. Physiol Cell Physiol 282, C595–C605 (2002). 11832345 10.1152/ajpcell.00270.2001

[b33] Gerum R. C., Richter S., Fabry B. & Zitterbart D. P. ClickPoints: an expandable toolbox for scientific image annotation and analysis. Methods in Ecology and Evolution, doi: 10.1111/2041-210X.12702 (2016).

