# Supplementary material for: The role of focal adhesion anchoring domains of CAS in mechanotransduction
Source: Sci Rep. 2017 Apr 13;7:46233. doi: 10.1038/srep46233 (PMC5390273; doi:10.1038/srep46233)

Supplemental Material for the Manuscript:

The role of focal adhesion anchoring domains of CAS in mechanotransduction

Jaroslav Braniš[1], Csilla Pataki[1], Marina Spörrer[2], Richard Gerum[2], Astrid Mainka[2], Vladimir Cermak[1], Wolfgang H. Goldmann[2], Ben Fabry[2], Jan Brabek[1] and Daniel Rosel[1]

[1] BIOCEV at Faculty of Science, Charles University in Prague, Vestec, Czech Republic
[2] Biophysics Group, Department of Physics, University of Erlangen-Nuremberg, Erlangen, Germany

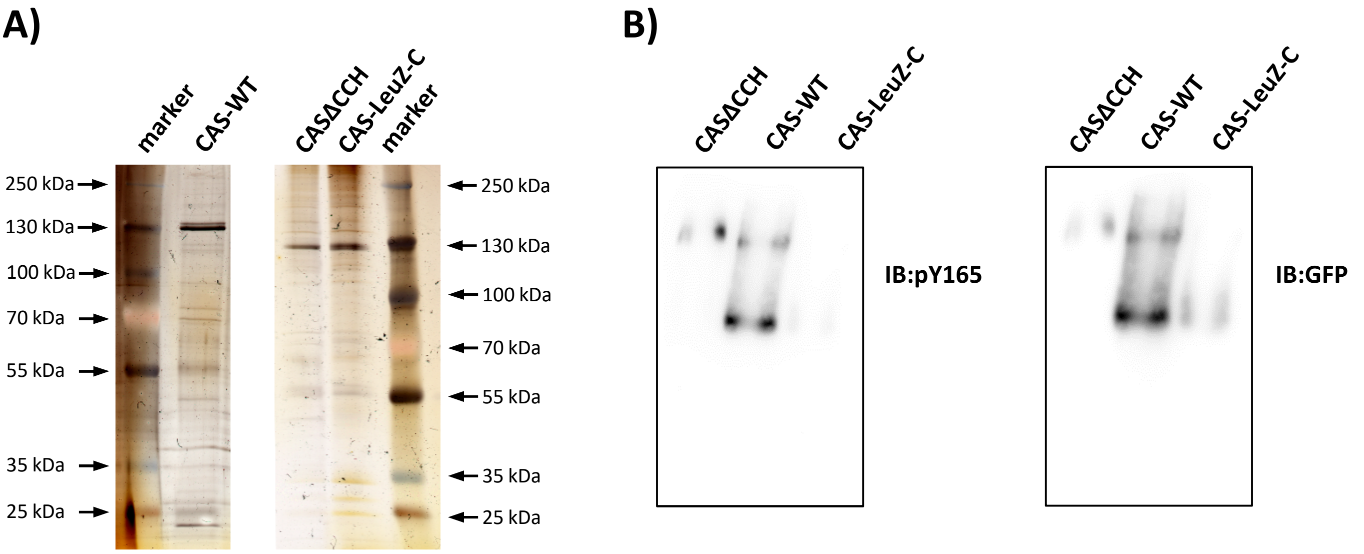

Supplemental Figure S1: CCH domain mediates CAS dimerization. The CAS-/- cells were transfected in transiently expressed indicated GFP-fused CAS variants. A SDS-PAGE of immunoprecipitated GFP-fused CAS variants (Silver staining). B Immunoblot analysis of immunoprecipitated GFP-fused CAS variants separated by native PAGE and subsequently detected by anti-P-CAS (left) and anti-GFP antibody (right).

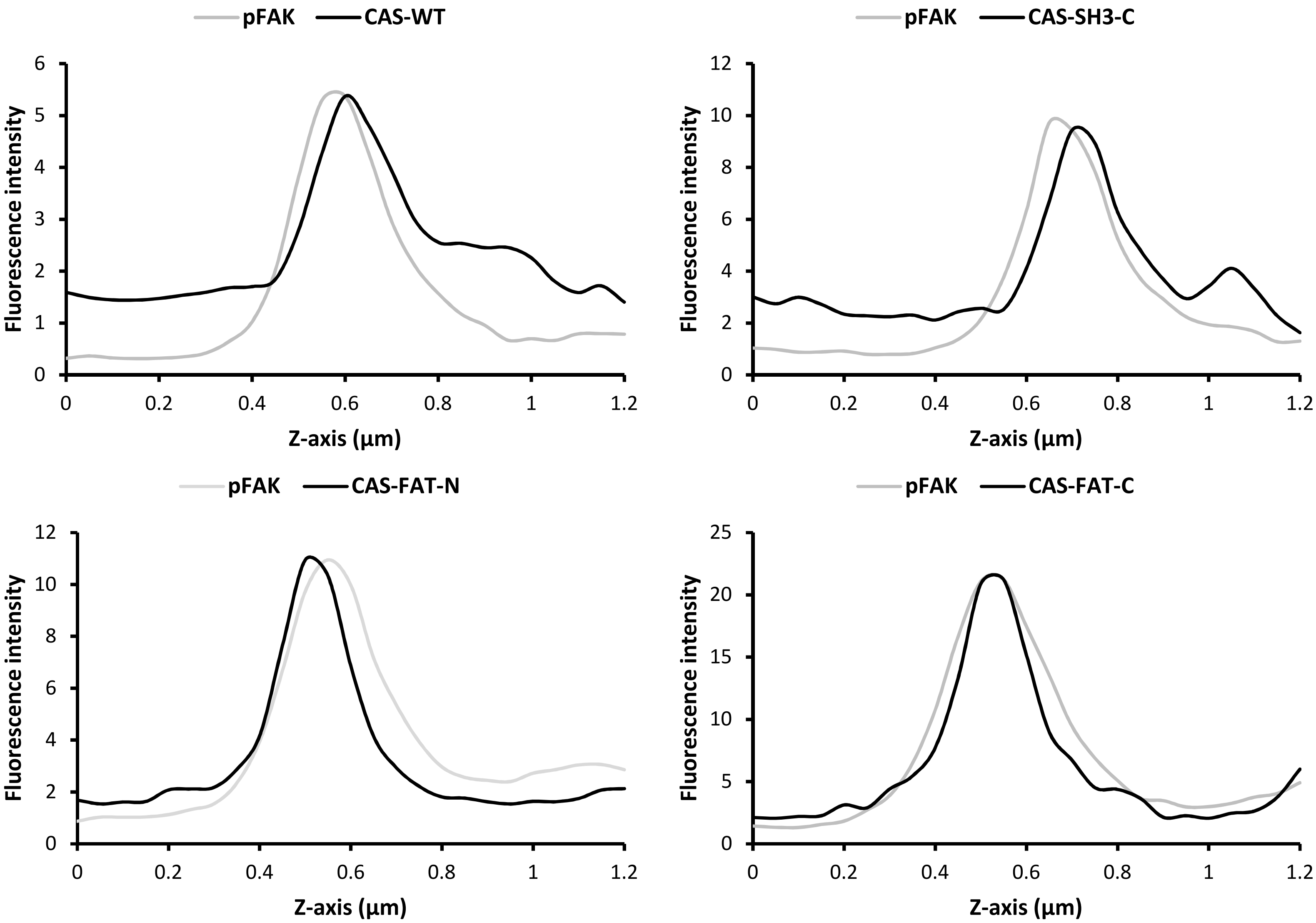

Supplemental Figure S2: Nanoscale localization of CAS variants in focal adhesions. CAS -/- cells stably expressing GFP fused CAS variants were analyzed for vertical distribution of fluorescence intensity signal of CAS variants and phospho FAK (pFAK) in focal adhesions (representative graphs).

Uncropped immunoblots

Figure 1B

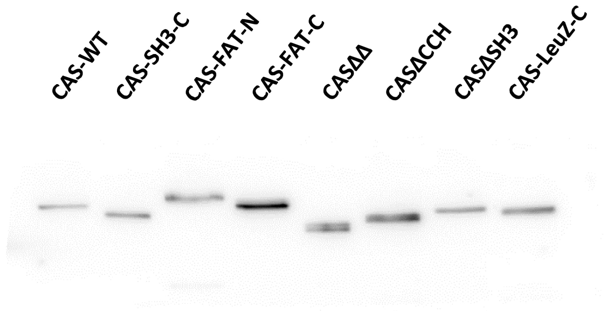

Figure 4A

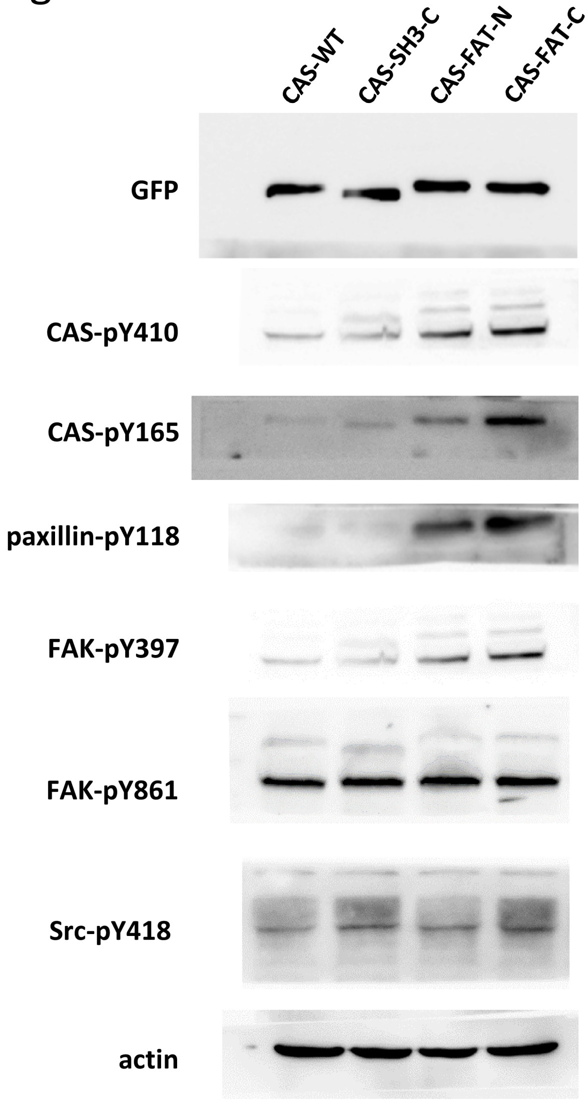

Figure 4B

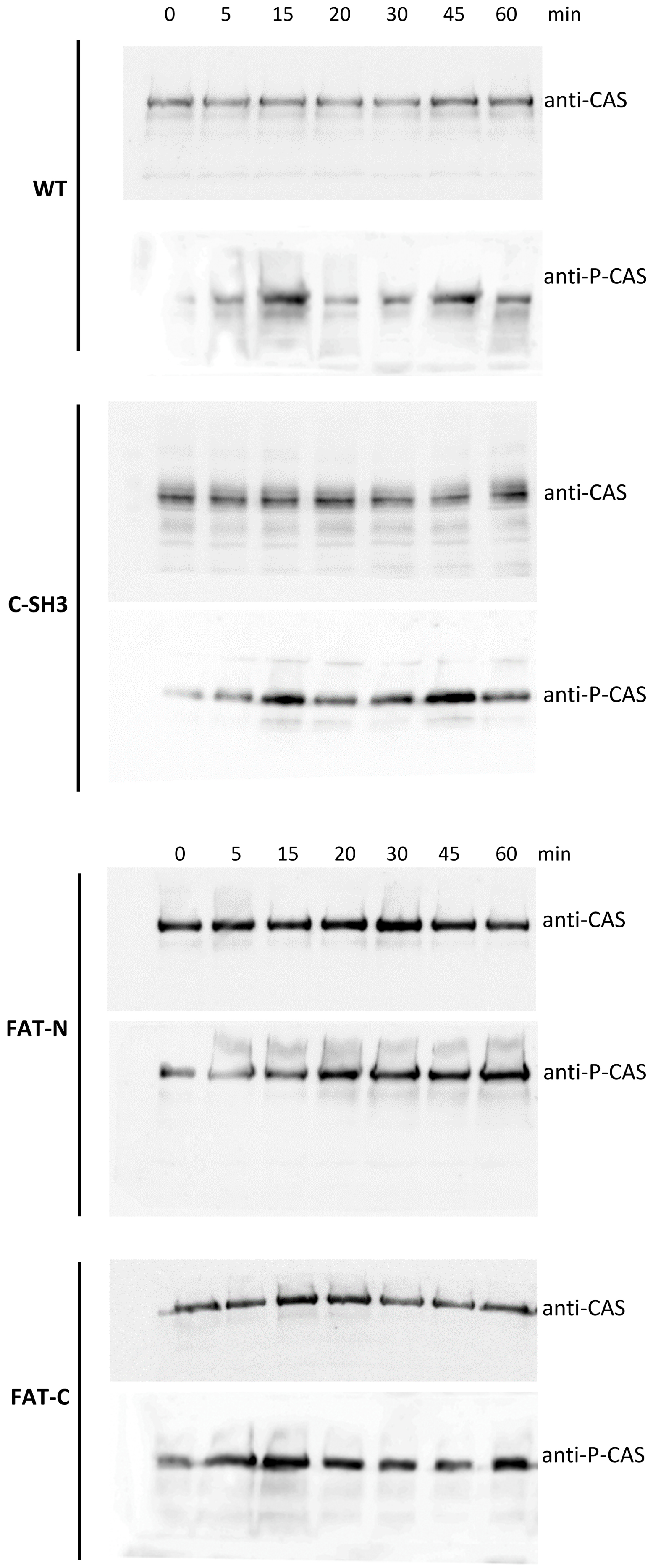

Supplement: Supplementary Information [file srep46233-s1.pdf]
